# Measuring aniseikonia and investigating neuroplasticity and image factors in amblyopia (MAGNIFY): study protocol for a randomised clinical trial

**DOI:** 10.1186/s13063-022-06159-2

**Published:** 2022-04-27

**Authors:** Jayshree South, Tina Gao, Melinda Calderwood, Jason Turuwhenua, Paul Roberts, Arier Lee, Andrew Collins, Joanna Black

**Affiliations:** 1 School of Optometry and Vision Science, The University of Auckland, Auckland, New Zealand; 2 Auckland Bioengineering Institute, The University of Auckland, Auckland, New Zealand; 3 School of Optometry and Vision Science, The University of Auckland, Auckland, New Zealand; 4 Medlink Innovation Limited, Auckland, New Zealand; 5 Section of Epidemiology and Biostatistics, The University of Auckland, Auckland, New Zealand

**Keywords:** Aniseikonia, Anisometropia, Amblyopia, Iseikonic, Subjective aniseikonia

## Abstract

**Background:**

Aniseikonia represents a potential barrier to neuroplasticity which may limit visual outcomes in children with anisometropic amblyopia. Full correction of refractive error is the first step in standard amblyopia treatment, which corrects for image focus but neglects image size differences.

**Methods:**

The MAGNIFY study is a double-masked, randomised clinical trial investigating the effectiveness of aniseikonia correcting lenses in children at first diagnosis of significant anisometropia. We hypothesis that aniseikonia correction lenses will improve image clarity and reduce the retinal size differences producing better visual acuity and stereoacuity improvements after 15 weeks of optical treatment for children with anisometropia. Eligible children will be randomly allocated to the treatment group (aniseikonia-correcting spectacle lenses) or control group (standard spectacle lenses). Visual acuity and binocular functions will be assessed every 5 weeks during the 15-week optical treatment phase according to standard amblyopia treatment protocol.

**Discussion:**

It is possible that correcting aniseikonia along with anisometropia at first diagnosis will promote binocularity as well as increase spectacle adherence by reducing visual discomfort, improving optical treatment outcomes. This could then reduce the need for additional amblyopia treatment such as patching or atropine, reducing the burden on hospital eye departments and potentially improving visual outcomes for children with amblyopia.

**Trial registration:**

Australian New Zealand Clinical Trials Registry (ANZCTR) ACTRN12620000061932. Registered on 24 January 2020. Protocol 15th November 2019, version one.

**Supplementary Information:**

The online version contains supplementary material available at 10.1186/s13063-022-06159-2.

## Background and rationale

Amblyopia is a developmental disorder of vision that affects approximately 3–5% of the population resulting in bilateral and unilateral visual impairment. Unilateral amblyopia arises from unequal input during early visual development commonly caused by misalignment of the eyes (strabismus), large differences in refractive error between eyes (anisometropia) or a disruption of the visual axis (form deprivation). About two thirds of children with amblyopia have anisometropia alone or in combination with strabismus [1–3]. Refractive correction alone (“optical treatment”) is the first-line treatment for amblyopia in children [4, 5], and this can successfully resolve amblyopia (interocular difference of ≤ 1 line) in about 30% of children [6, 7]. The remaining 70% of children usually undergo occlusion or atropine penalisation therapy to further improve vision in the amblyopic eye.

Anisometropia is usually caused by the two eyes having different axial lengths or by the refractive components of the eyes (cornea, lens) being different in shape. Based on optical theory, these differences between eyes can cause the retinal images to be of different size [8–10], a phenomenon known as aniseikonia. Aniseikonia can also be induced optically by spectacle lenses placed in front of the eye, as the power of the lens has an effect on both the clarity and size of the retinal image [11]. This means that patients with anisometropia are likely to experience aniseikonia, and that spectacle corrections for their refractive error can sometimes worsen the aniseikonia [12]

In adults, induced aniseikonia can interfere with binocular functions like stereoacuity and cause visual discomfort [13]. However, in children with anisometropia, the current standard clinical prescribing guidelines do not account for image size differences. Many clinicians usually prescribe standard spectacle lenses that fully correct anisometropia and assume that children will adapt to aniseikonia without any detrimental effect on their binocular vision or visual gains during optical adaptation. Theoretically, significant aniseikonia may also hinder stereoacuity in children, potentially limiting treatment effectiveness. Aniseikonia is potentially responsible for the plateauing of vision often found during standard treatment protocols [14, 15] and the reduced stereoacuity [16] which often results.

Aniseikonia can be corrected by altering the base curve, thickness, and refractive index of spectacle lenses to alter its magnification effects. This can be done in such a way to reduce the image size difference between the two eyes. A number of companies currently supply lenses designed to correct aniseikonia, and these iseikonic lenses are often used for patients with anisometropia (with or without amblyopia). However, there has not been a direct comparison study investigating the effectiveness of these aniseikonia-correcting lenses compared to standard spectacle lenses for treatment of anisometropic amblyopia in children.

## Objectives and hypothesis

### Research hypothesis

Aniseikonia correction lenses will improve image clarity and reduce the retinal size differences producing better visual acuity and stereoacuity improvements after 15 weeks of optical treatment for children with anisometropia.

### Study objectives

#### Primary objective

The primary objective is as follows: to assess the change in visual acuity of the amblyopic eye from baseline after 15 weeks of wearing spectacle lenses that equalise retinal image size as well as retinal image clarity in children with anisometropic amblyopia. We hypothesise that correction of aniseikonia will help binocularity, producing greater visual acuity and stereoacuity improvements after 15 weeks of optical treatment.

#### Secondary objectives

The secondary objectives are as follows: change from baseline in stereoacuity after 15 weeks of spectacle wear. Improvement in stereoacuity is a key secondary objective as correcting aniseikonia may reduce the binocular mismatch of image size, allowing improved binocular combination and stereopsis.

Objective adherence with optical correction will also be assessed. Iseikonic corrections may improve spectacle comfort in those with anisometropia, which may then lead to better spectacle adherence and in turn, better visual outcomes.

## Methods

### Study design

The MAGNIFY study is a randomised clinical trial, designed as a superiority study with investigator and participant masking to treatment allocation, investigating the visual acuity and stereoacuity outcomes of incorporating aniseikonic correction (lenses that equalises image size and image clarity) into spectacles for children with anisometropic amblyopia compared to standard lenses which correct for image clarity only. This protocol was developed according to SPIRIT guidelines [17].

### Setting

Data will be collected at the Optometry Clinic at the School of Optometry and Vision Science, the University of Auckland, Grafton Campus, Auckland.

### Participants and recruitment

Potential participants will be identified through referrals from paediatric ophthalmologists and experienced paediatric optometry clinics which follow the same prescribing guidelines for children. Initial diagnosis of anisometropia will be made following a comprehensive eye examination including binocular function testing and a cycloplegic refraction. Potentially suitable participants/parents/guardians will be given a copy of the participant information sheet by their eye care provider. With consent from the patient/parents/guardians, the eye care provider will send the contact information to a member of the study team who will contact the participant and invite them to a registration visit to assess eligibility. Parents/guardians of potential participants are also able to contact the study team directly and a copy of the participant information sheet will be sent either electronically or by mail and consent will be sought to contact their eye provider for release of the relevant clinical information. The registration visit will be used for the consenting procedure and for assessing eligibility (Appendix 2).

Recruitment began on 27 February 2020 and is scheduled to end 11 October 2022.

### Eligibility

The following inclusion criteria exist for this trial:

- Children aged 4 to < 8 years old
- Anisometropia that has not yet been treated by spectacles or occlusion
- Uncorrected visual acuity in the worst eye of 6/12 (0.30 logMAR) or worse, with an interocular difference of 2 lines or more. Sound eye must be 6/7.5 (0.10 logMAR) or better, with an interocular difference of 2 lines or more
- Anisometropia ≥ 1.50 DS difference in spherical equivalent between eyes as determined by cycloplegic refraction
- Astigmatism ≤ 3.00 DC
- No manifest strabismus at near or distance on cover test
- Healthy eyes
- Willing and able to wear spectacles full-time

The exclusion criteria are as follows:

- Myopia exceeding -6.00 DS in spherical equivalent
- Ocular pathology such as a congenital cataract (aphakia/pseudophakia), retinopathy of prematurity, keratoconus
- Previous eye surgery of any kind
- Any known neurological conditions that could potentially affect vision
- Contact lens wear

### Optical treatment

Optical correction is prescribed based on a cycloplegic refraction that is not more than six months old. The correction will be prescribed using criteria designed by the Paediatric Eye Disease Investigator Group [18] and must be as follows: *Hypermetropia—*not under-corrected by more than + 1.50D spherical equivalent and the reduction in plus sphere must be identical between the two eyes. *Anisometropia—*full correction of the anisometropic difference. *Astigmatism—*full cylinder power prescribed. *Myopia—*full correction of myopia. If the participant does not have a copy of a recent (< 6 months old) cycloplegic refraction from their eye care provider, they will be asked to undergo a comprehensive eye examination including a cycloplegic refraction at the University of Auckland Optometry clinic. This will be used to confirm eligibility prior to being enrolled into the study.

### Variable definitions

#### Visual acuity

Amblyopia impairs a broad range of visual functions in the affected eye including visual acuity [19], contrast sensitivity [20] and motion perception [21, 22]. As visual acuity is the gold standard measure of visual function in clinical settings, a widely accepted clinical definition of amblyopia is a difference in best corrected visual acuity between the two eyes of 0.20 log units in the presence of an amblyogenic factor and the absence of any ocular or optic nerve pathology [19]. The amblyogenic factor of interest in this study is anisometropia of ≥ 1.50 DS difference in spherical equivalent. Distance visual acuity (VA) measures are collected separately for OD (RE) and OS (LE). Distance visual acuity will be measured using the highly standardised HOTV protocol adopted by the Amblyopia Treatment Study group and recorded as logMAR units [23].

#### Stereopsis

Amblyopia is commonly associated with impaired stereoscopic depth perception under ordinary (binocular) viewing conditions [24]. Stereopsis will be measured using the clinically well-established Randot Preschool test (Stereo Optical Co. Inc., Chicago, IL, USA) [25]. Stereopsis will be analysed as a categorical outcome: “Improved” or “Not improved” at each follow-up compared to the baseline visit. The participant’s stereopsis threshold is considered to have improved compared to baseline if there is a reduction in log10 (Threshold) value of 0.60 (2-octaves) or more [26] or if the participant’s result changes from Nil Stereo to a measurable threshold. Stereopsis is considered to have worsened compared to baseline if there is an increase in log10 (Threshold) of 0.60 or more or if the participant’s result changes from a measurable threshold to Nil Stereo.

#### Subjective aniseikonia

Aniseikonia is likely to be present in those with anisometropic amblyopia [27, 28]. The difference in images sizes could potentially hinder binocular vision. Currently, estimations [29, 30] or empirical calculations [31, 32] are used due to presumed difficulties in measuring subjective aniseikonia, especially in children. Estimations and empirical calculations, however, do not take into account the retinal and cortical adaptations that may occur and therefore accurate measurement of subjective aniseikonia is important to determine the amount of image size difference individually experienced. Subjective aniseikonia is difficult to measure in adults and children, and currently, there is no gold standard test available, and no tests are specifically designed for use in children. A previous study successfully used the Aniseikonia Inspector Version 3 [33] in school-aged children (5 to 13 years old). This current study uses two validated and commercially available tests: the Aniseikonia Inspector Version 3 (Optical Diagnostics, Culemborg, The Netherlands) and the New Aniseikonia (Awaya) Tests (Good-Lite Company, Tokyo, Japan) [34]. These tests will be used to examine testability in pre-school (4– < 8 years old) children in the presence of anisometropic amblyopia.

#### Spectacle adherence

Spectacle adherence is correlated with visual improvements during optical treatment of amblyopia. A wide range of inter-individual variability, with a potential dose-response relationship between visual improvements and hours of spectacle wear, has been previously shown [35]. Currently, adherence with spectacle wear is only assessed indirectly and subjectively via parental reporting [36, 37], which is generally expected to overestimate adherence. This study will include an optional custom-built objective monitoring device (SpecsOn monitor) [38] to monitor spectacle adherence alongside a daily spectacle wear diary for the first 5 weeks. Additional consent for the SpecsOn monitor will be provided on the consent form at the enrolment visit (Appendix 1). The SpecsOn monitor is a small device externally mounted on the spectacle arm and incorporates two temperature sensors. One sensor, directed at the wearer’s temple, measures skin temperature using an infrared detector, the other sensor measures the device’s temperature as an estimate of the ambient temperature. Temperature measurements are taken at 5-min intervals and written to non-volatile memory. Comparisons of the two temperature measurements can be used to determine if spectacles are being worn. The SpecsOn device will be removed after a minimum of 5 weeks, and the glasses wear diary will be continued for the full 15-week duration. As not all participants will opt to wear the SpecsOn monitor, the glasses wear diary will be the main source for adherence monitoring data. Spectacle wear compliance will be based on the total time the participants wore their glasses as recorded in the participants daily wear diary. Diary recording will be encouraged at every study visit.

- A participant is considered compliant if they have worn their glasses for ≥ 75% of their awake time. Awake time is estimated by parents and recorded at the registration visit.

#### Registration and enrolment visit

The registration visit will be used to screen, assess eligibility and enrol participants that meet the eligibility criteria. Demographic data, ocular and medical health history and biometry using the LenStar LS 900 will be taken. Biometry data will be used to determine the basis of anisometropia. Optical Coherence Topography Scans of the macular (3D macula: 7 × 7 mm) and retinal nerve fibre layer (3D Optic Disc 6 × 6 mm scan) will be taken using the DRI Triton Optical Coherence Topographer to confirm eye health. Cover testing is performed at near and distance to screen for strabismus, but visual acuity, fusional vergence/peripheral fusion, ocular motility and stereopsis will only be assessed if this information is not available from the referrer. Eligible participants will then be randomly allocated to either the control or the treatment group.

#### Randomisation

Participants will be randomised to the control standard lenses group or treatment aniseikonia-correcting lenses group after they are confirmed to be eligible for the trial and have consented to take part. Allocation to each group will at a 1:1 ratio using a computer-generated randomisation schedule [39] by an unmasked study member. The schedule was generated using randomised permuted blocks with fixed block sizes. The block sizes will not be disclosed to ensure concealment. Unmasked study staff will perform spectacle dispensing to ensure correct fitting and make all spectacle adjustments required at data collection visits.

### Appointment schedule

#### Baseline visit

The baseline visit is when participants collect their new spectacles. This visit contains the first aided visual acuity and binocular function assessment. A minimum of 15 min of adaptation time to the new spectacles will be given before vision testing is commenced. To allow adaptation time, testing is to be carried out in the following order:

1. Dispensing and spectacles fitting by unmasked examiner
2. Parent and child to complete PedEyeQ questionnaire
3. Spectacle adherence diary given and explained
4. Distance visual acuity test using the EVA tester
5. Cover test with refractive correction in place at 33 cm and 6 m
6. Cover test without refractive correction at 33 cm and 6 m
7. Ocular motility
8. Horizontal fusional vergence range test using prism fusion range at 33 cm and 6 m.
  If horizontal fusional vergence range testing is not possible, then peripheral fusion will be assessed using the 20Δ base out test in front of either eye. If a 20Δ prism is not overcome, then a 10Δ base out prism will be used.
9. Vertical fusional vergence range test using the prism fusion range at 6 m
10. Stereopsis using the Randot Preschool stereotest
11. Aniseikonia Inspector V3
12. The New Aniseikonia Test (Awaya)

The PedEyeQ questionnaire will be used to assess functional vision and eye-related quality of life (ER-QOL) in children and their parents [40]. Following the baseline visit, participants will be asked to wear their spectacles full-time until they have completed 15 weeks of spectacle wear.

#### 5-week, 10-week and 15-week follow-up visits

Participants will be required to attend three 20-min follow-up visits, at 5, 10, and 15 weeks from baseline. At each follow-up visit, a research orthoptist masked to the child’s treatment allocation will assess visual acuity, stereopsis, ocular alignment, near point of convergence, horizontal fusional vergence range or 20Δ base out test for peripheral fusion, bifoveal fixation using the 4 Δ base out reflex test and visuoscopy. Subjective aniseikonia will be reassessed at the 15-week follow-up visit only. Adherence with spectacle wear will be monitored by completing a daily spectacle wear diary which will be checked and encouraged at each follow-up visit. Objective monitoring with the SpecsOn spectacle-mounted adherence monitor will be removed after a minimum of 5 weeks. However, if participants are willing, this may be extended to 10 weeks.

#### Intervention

Participants are allocated to one of two groups, either the control group (standard spectacles) with lenses that correct only refractive error, or the treatment group (iseikonic spectacles) where lenses incorporate aniseikonia correction as well as refractive correction. Participants will be provided with spectacles at no cost to them for fulltime wear for 15 weeks and will be able to keep these spectacles after study participation. Aniseikonia correction will be provided using SHAW lenses (Canada) (https://shawlens.com) through the local Australasia distributor, CR Surfacing labs. Standard amblyopia Shaw lens ordering procedures will be followed. Iseikonic lenses are sometimes used in optometry practices in New Zealand and overseas for optical correction in children and adults with anisometropia to help reduce visual discomfort and aid in adaptation. The optional objective spectacle monitoring device [38] will be fitted to the spectacle frames and parents of all participants will be asked to complete a daily spectacle wear diary to monitor adherence.

#### Intervention modifications and adherence

Asthenopia can result from adaptation to new lenses and may initially cause some discomfort, but this should improve within the first few weeks and persistence with the spectacles will be encouraged. Adherence to spectacles will objectively be monitored for the first 5 weeks, but participants will fill out a daily spectacle wear diary for the full 15 weeks where they will be required to record times when spectacles were removed, the duration without spectacles and the activities that required them to remove the spectacles. Diary entries will be checked and encouraged at all follow-up visits. No other concomitant therapy for amblyopia or alternative refractive correction is allowed for the full 15-week period.

#### Masking

The clinical examiner, participants and their parents will remain masked to treatment allocation throughout the whole study. Personnel involved in recruitment, data collection (assessing the outcomes) and those involved with data entry, data cleaning and analysing the results will also be masked until the code is broken after data lock. Masked clinical examiners may be able to identify treatment allocation if they look closely at the spectacles. Therefore, masked clinical examiners will not handle the spectacles at all. Any adjustments required to the frames or assessment of repairs will be performed by unmasked study staff, who do not conduct any clinical examinations. Participants and parents/caregivers attending visits will also be instructed that the masked clinical examiner will not be able to answer any questions about their lenses. Participants will be informed about their group allocation by letter/report once all data has been collected and analysed.

#### Study schedule

**Fig. 1:**
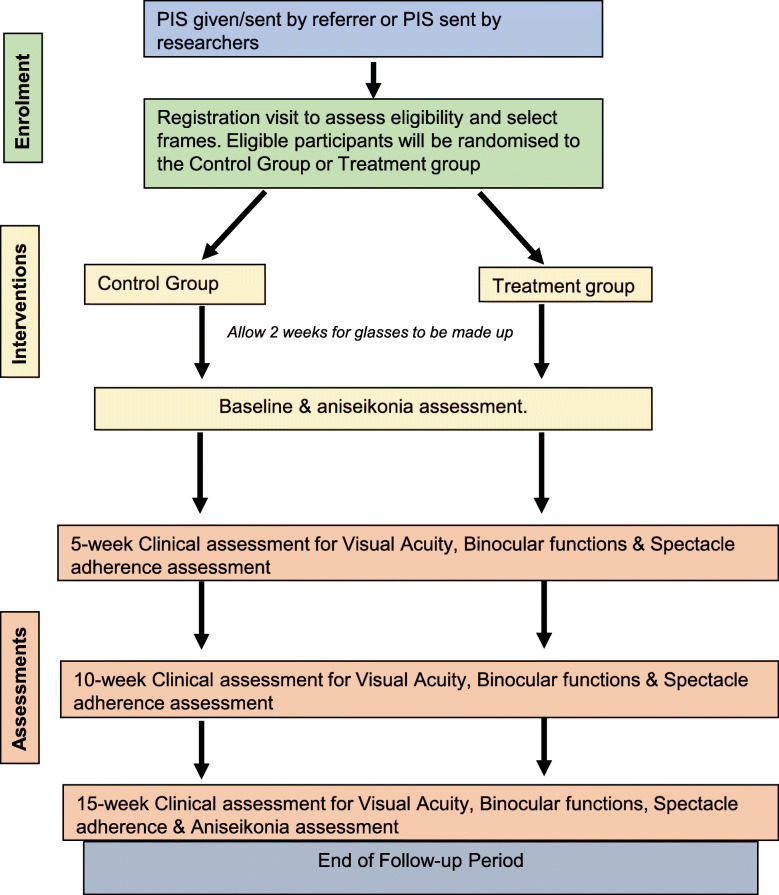
Schematic summary of the MAGNIFY schedule of enrolment, interventions, and assessments

The study schedule has been summarised in Fig. 1.

### Data collection

#### Outcome

Outcome measures will be assessed at 5-, 10-, and 15-week post-randomisation by a masked clinical examiner. All study members will be trained on study requirements. Masked clinical examiners will use standard clinical measurements to complete paper-based data collection forms designed by study investigators. Baseline and outcome measures are summarised in Table 1. A trial management team will be checking the integrity of data collected on the paper forms as they are entered onto the database to ensure data quality and completeness.

**Table 1:** Schedule of tests and follow-up visits

Measures	Registration	Baseline assessment	5 weeks	10 weeks	15 weeks
Demographics	✓				
Visual acuity	✓^a^	✓	✓	✓	✓
Ocular alignment	✓	✓	✓	✓	✓
Motor fusion	✓^b^	✓	✓	✓	✓
Stereopsis	✓^b^	✓	✓	✓	✓
Subjective aniseikonia tests		✓			✓
Biometry	✓				
OCT	✓				
PedEyeQ		✓			✓
Serious adverse events			✓	✓	✓
Spectacle adherence			✓	✓	✓
Allocation	✓				

^a^Unaided visual acuity is measured at the registration visit if not available from the referrer. The Keeler LogMAR chart will be used in the study for those who require measurement at the registration visit

^b^Tests done by study clinicians only if relevant information is not available from the referrer

#### Primary outcome

The primary outcome measure will be change in amblyopic eye VA from baseline to the 15-week outcome visit, measured using the highly standardised HOTV protocol on the electronic visual acuity (EVA) testing system [23].

#### Secondary outcomes

- Change from baseline in distance visual acuity of the amblyopic eye at 5- and 10-week post baseline assessment
- Change from baseline in distance visual acuity of the fellow eye at 5, 10 and 15 weeks
- Change from baseline in interocular acuity difference at 5, 10 and 15 weeks
- Change from baseline in stereopsis at 5-, 10- and 15-week follow-up visits, measured using the highly standardised Randot Preschool stereotest [25]
- Spectacle wear adherence at 5-, 10- and 15-week visits, measured using the daily spectacle-wear diary
- PedEyeQ quality of life metric questionnaire to assess functional vision and eye-related quality of life (ER-QOL) in children and their parents [40]

### Participant retention

Once a child is randomised, study staff will make every possible effort to follow the child for the entire study period. Reminders of upcoming study visits will be sent via the preferred method of communication indicated at the enrolment visit and contributions will be made towards travel costs for each visit.

### Conclusion of the study

At the end of the 15-week study period, all participants will be referred back to the eye care provider that referred them with a full report of their status. If vision in the amblyopic eye has not sufficiently improved after 15 weeks of fulltime spectacle wear, further amblyopia treatment (such as patching therapy) will be provided by referral to the appropriate eye care provider. Any adverse events to the participant and/or spectacles will be monitored and recorded as appropriate.

### Sample size

A sample size of 50 patients (25 per arm) will provide 95% power at *p* = 0.05 to detect a minimal clinically important difference of 0.20 log units improvement in visual acuity as tested on the EVA test [41, 42] at 15 weeks, assuming a standard deviation of 0.17 [43]. This sample size allows for an overall loss-to-follow-up of 15% [44] (calculated using GraphPad Sat Mate 2.0).

### Data management

All participants will be assigned a unique identification code to protect confidentiality immediately following data collection. All clinical data will be collected, recorded, stored and analysed under this unique research code. All paper-based clinical documents will be safely stored in a locked secure cabinet at the School of Optometry and Vision Science for 6 years before being securely destroyed. De-identified electronic data will be stored indefinitely on password-protected computers to allow comparison to future data sets. Consent forms and referral letters, which contain identifying information, will be held in a secure location, separate from the research data for a period of 6 years.

### Data analysis

Statistical analyses will be performed using SAS version 9.4. All statistical tests will be two-tailed and at a 5% significance level throughout the analyses, and all treatment evaluations will be performed on the principle of ‘intention to treat’ unless otherwise specified. The ITT population will consist of all randomised participants according to their randomised group, regardless of the treatment actually received and subsequent withdrawal or deviation from the protocol. People who subsequently withdrawal from the study will contribute their data already collected in the ITT analysis. A per protocol analysis will also be performed on the primary outcome to assess the robustness of the results. Randomised participants who have no major protocol violations will be included in this subset for analysis. Relevant protocol violations may include errors in treatment assignment, the use of excluded medication, poor compliance, loss to follow-up and missing data. For this trial, possible conditions of major protocol violation include:

1. Poor spectacle wear compliance (< 75% of waking hours)
2. Loss to follow-up and missing outcome
3. Follow-up visits fall out of time window: out of ± 7 days for 5-, 10- and 15-week follow-up visits

The study investigators will conduct a blinded review of all participants with protocol violations before final data analysis and make decision on the list to be excluded from the per protocol set. Participants that withdraw will contribute data to the point of withdrawal unless data withdrawal is specifically requested. No adjustments for multiple comparisons are planned for any of the outcomes. No imputation will be performed for missing data.

All results will be presented overall and by treatment groups. Summaries of continuous variables which are normally distributed will be presented as means and standard deviations or medians and inter-quartiles for skewed data, while categorical variables will be presented as frequencies and percentages.

For logMAR visual acuity, smaller or more negative values equal better vision. For this study, change from baseline (0 week) to follow-up in distance visual acuity of the amblyopic eye will be calculated as (baseline–follow-up). Positive change from baseline values indicates there is improvement, and negative values indicate worsening of the condition.

When the method of mixed model for repeated measure (MMRM) is used, the model will include treatment, time, treatment by time interaction, baseline value as fixed effects and subject as random effect, unless otherwise stated. The within-subject errors will be modelled using an appropriate covariance matrix. Candidate structures include but are not restricted to unstructured, autoregressive, Toeplitz, compound symmetry and spatial. Kenward-Roger method will be used to estimate the denominator degrees of freedom for fixed effects.

The results will be published in journals and through conference presentations to the relevant professional groups. Participants can choose to receive a summary of the overall trial results by letter/report once all data has been collected and analysed. This is an option on the consent form.

### Data monitoring

An independent monitor will check the existence and correct date for all signed consent forms. The monitor will also sample over 10% of all randomised participants to check accuracy of data on the database against source data. This trial is considered to be low risk and, therefore, establishing a Data Safety Monitoring Committee for the trial is not necessary.

Interim analysis is performed when 50% of the patients have been randomised and completed the 15 weeks follow-up. The interim analysis is performed by an independent statistician, masked to treatment allocation.

### Harm

We do not expect any adverse effects to arise from the lenses in this study, as both types of lenses are currently used in New Zealand and overseas in the treatment of anisometropic amblyopia in children. Normal spectacle lenses have been shown to resolve amblyopia in 30% of children and no adverse effects have been reported [6, 7, 45]. Aniseikonia lenses have been found to reduce asthenopic symptoms (headaches/eyestrain/blurry vision) that are normally associated with adapting to a new prescription in adults [46], and there have not been any reports of adverse effects from clinical use in children.

Adverse events unrelated to the study treatment may occur, e.g. circumstances that prevent the child wearing their glasses such as illness or broken glasses. These events will be documented and monitored closely throughout the clinical trial.

All adverse events and spectacle related incidents occurring during the trial and that are observed by study personnel or reported by the participant will be recorded, whether or not attributed to trial treatment. Adverse events will be collected systematically at each follow-up visit and open-ended questioning will encourage participants to report on unexpected adverse events. Those that are considered related to the trial treatment as judged by a qualified investigator will be followed either until resolution or the event is considered stable. It will be left to the judgement of the qualified study investigators to decide whether or not an adverse event is of sufficient severity to require discontinuing the participant from the study. A participant may also voluntarily withdraw from participating in the study due to what he or she perceives as an intolerable adverse effect. If either of these occurs, the participant will undergo follow-up visits for trial assessment and be given appropriate care under medical supervision until symptoms cease, or the condition becomes stable.

An independent monitor will review the source documents (paper data collection forms) and determine whether the data reported in the database system are complete and accurate. Quality assurance of the lenses and spectacles will be undertaken by the unmasked team members that are responsible for ordering lenses and quality checking them for correction prescription and lens allocation.

### Ethical considerations

Ethical approval has been granted by the University of Auckland Human Participants Ethics Committee (023628). Assent will be sought prior to study enrolment from all children. Written informed consent will be signed by the parent/guardian of the child before any data collection procedures. All participants (or parents/guardians of participants) may withdraw at any time from the study (Appendix 3).

### Protocol amendments

Any modifications to the protocol which may impact on the conduct of the study, potential benefit of the patient or may affect patient safety including changes of study objectives, study design, patient population, sample sizes, study procedures or significant administrative aspects will require a formal amendment to the protocol. Such amendment will be agreed upon by MAGNIFY study group and approved by the University of Auckland Human Participants Ethics Committee prior to implementation*.*

### Consent and assent

Participant’s caregivers and children themselves will be given participant information sheet prior to the enrolment visit (Appendix 2 and 4). Both children and care givers will be encouraged to ask questions and have an informed discussion on what is involved with study participation. The research orthoptist will then obtain written consent from the caregivers and assent will be sought from children prior to collection of any data. There are no current plans for using data collected in this study in future studies.

### Confidentiality

All participants will be assigned a unique identification code at enrolment to protect confidentiality. All clinical study data will be collected, recorded, stored and analysed under this unique research code. A document linking the code with the participant’s name will be stored independently of the clinical data and will be available only to the researchers. This linking document will be destroyed after 6 years.

All clinical documents will be safely stored confidentially in a locked secure cabinet at the University of Auckland for 6 years before being securely destroyed. De-identified electronic data will be stored indefinitely on password-protected computers to allow comparison to future data sets. Consent forms will be held by the department in a secure location, separate from the research data for a period of 6 years.

## Discussion

This is the first double-masked, randomised clinical trial investigating aniseikonia-correction in optical treatment of anisometropic amblyopia. Significant aniseikonia is known to limit binocular functions such as stereoacuity in adults with normal visual history [47–49]; therefore, it is possible that aniseikonia also limits stereoacuity in children with anisometropic amblyopia. Correcting aniseikonia as part of the anisometropic correction may reduce the stimulus for developing suppression, leading to improved binocular visual outcomes from optical treatment of anisometropic amblyopia. There is currently no clinical trial-quality evidence to support or refute this hypothesis. Available evidence is limited to individual case reports where rigorous protocols have not been utilised [50].

Adherence to full time spectacle wear is essential for optimal visual outcomes. However, clinically, spectacle adherence is only assessed indirectly via subjective parental reporting which is generally expected to overestimate adherence [36, 37]. Current amblyopia research shows a wide variability in adherence to amblyopia treatments like occlusion [51–54] and parental over reporting of treatment adherence [55–57]. Adherence with spectacle wear also displays a similarly wide range of interindividual variability [35] and likely to suffer similar parental over estimation of adherence. In this study, the SpecsOn monitor [38] will be used alongside the daily spectacle wear diary to examine the effectiveness of the monitor in a clinical-like setting where children are reviewed routinely for amblyopia treatment. Not all participants will opt to have the device put onto their spectacles and therefore will not have complete data from all the enrolled participants in the study. However, data on objective assessment of adherence will contribute to the design and methodology of future studies of optical treatment.

Aniseikonia is likely to be present alongside anisometropia, as retinal image size difference caused by anisometropia itself and the spectacle lens induced magnifications from the optical correction of anisometropia both cause aniseikonia [9, 11, 58]. Retinal image size differences in anisometropia may be a contributing factor in stimulating suppression and the development of amblyopia [47–49]. Correcting image size difference alongside defocus in the treatment of significant anisometropia may further reduce the need to develop suppression and optimise visual recovery. However, subjective assessment of aniseikonia in preschool children with anisometropic amblyopia has not been reported on. It is presumed to be difficult due to potential suppression and the lack of binocularity required to complete direct comparison tests using the currently available tests. At present, there is no gold standard test for assessing subjective aniseikonia, with no tests designed for or validated in children. This study will use two commercially available tests to assess the reliability of measuring aniseikonia in preschool children with anisometropic amblyopia providing guidance for the design and methodology of future studies.

Providing aniseikonia corrections at first diagnosis of significant anisometropia may help us understand the importance of subjective aniseikonia measurements and its role in the treatment of anisometropic amblyopia.

## Trial status

This clinical trial was registered with the Australia New Zealand clinical trial registry (ANZCTR) on 24 January 2020 under registration number ACTRN12620000061932. Protocol version 1 was developed on 13 November 2019. Recruitment started January 2020 and is due to be completed by 11 October 2022 (Appendix 5).

## Supplementary Information

**Additional file 1: Appendix 1** Parental/caregiver consent form. **Appendix 2** Parent/caregiver participant information sheet. **Appendix 3** Child assent form. **Appendix 4** Child participant information sheet. **Appendix 5** Trial registration data set

## Data Availability

The named authors will have access to the final trial dataset and metadata will be shared through an appropriate data archive. Further data set will be available at request There are no contractual agreements that limit such access for investigators.

## Abbreviations

D: Dioptre
DS: Dioptre sphere
DC: Dioptre cylinder
EVA: Electronic visual acuity
ITT: Intention-to-treat
LogMAR: Logarithm of the minimum angle of resolution
OCT: Optical Coherence Topographer
ER-QOL: Eye-related quality of life
MMRM: Mixed model for repeated measure
VA: Visual acuity
RCT: Randomised clinical trial
ANZCTR: Australia New Zealand Clinical Trial Registry
