# Supplementary material for: Measuring aniseikonia and investigating neuroplasticity and image factors in amblyopia (MAGNIFY): study protocol for a randomised clinical trial
Source: Trials. 2022 Apr 27;23:358. doi: 10.1186/s13063-022-06159-2 (PMC9044861; doi:10.1186/s13063-022-06159-2)
Supplement: Supplementary file 1 — Additional file 1: Appendix 1 Parental/caregiver consent form. Appendix 2 Parent/caregiver participant information sheet. Appendix 3 Child assent form. Appendix 4 Child participant information sheet. Appendix 5 Trial registration data set [file 13063_2022_6159_MOESM1_ESM.docx]

# Appendices

## Parental/ Caregiver Consent Form


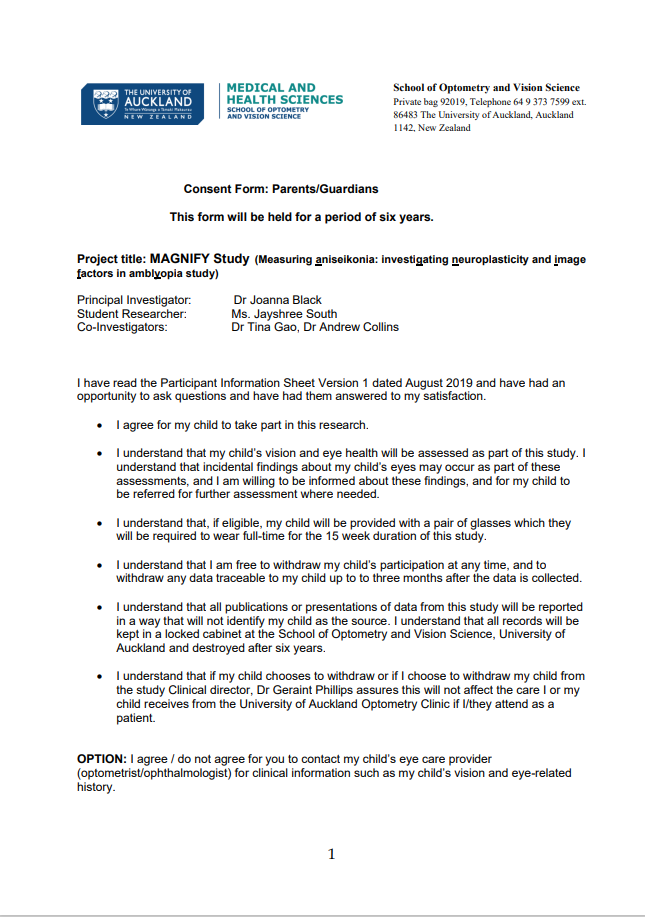


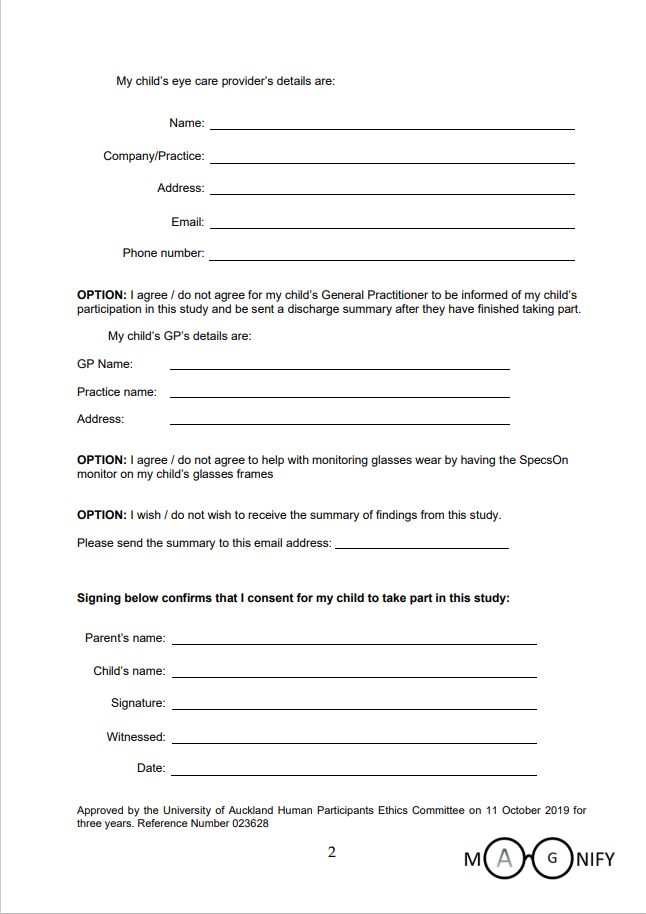


## Parent/Caregiver Participant Information Sheet


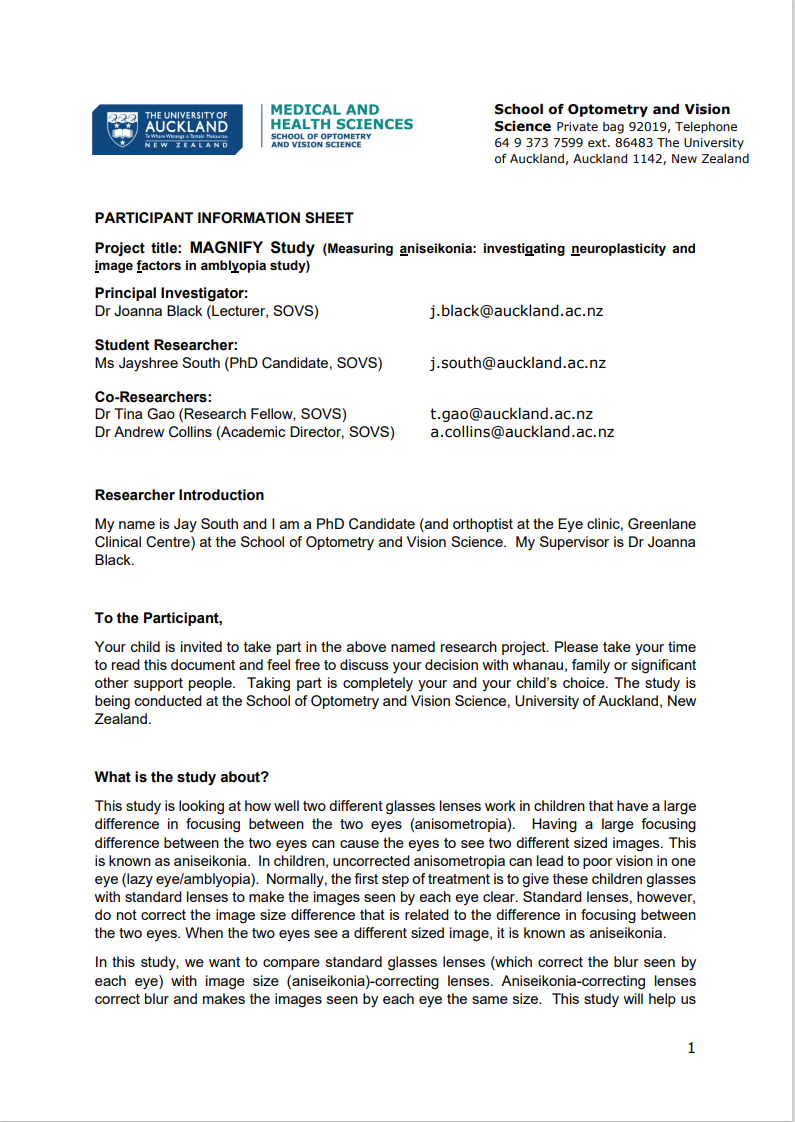


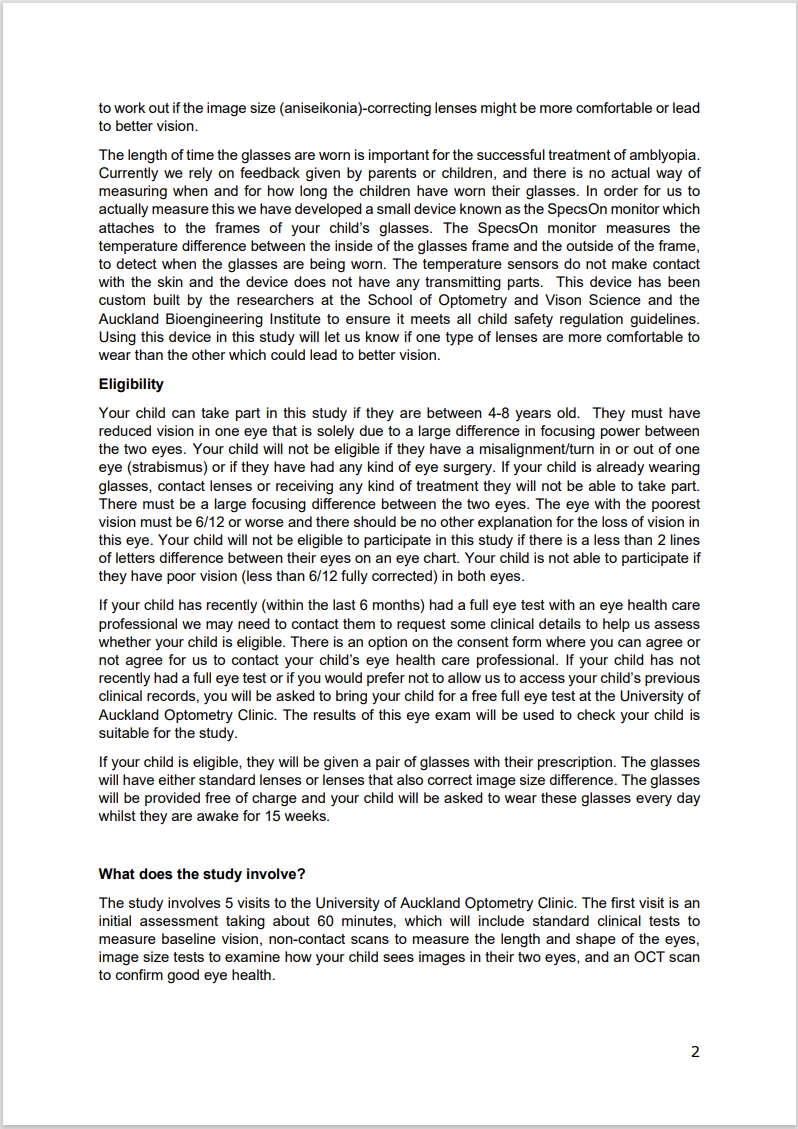


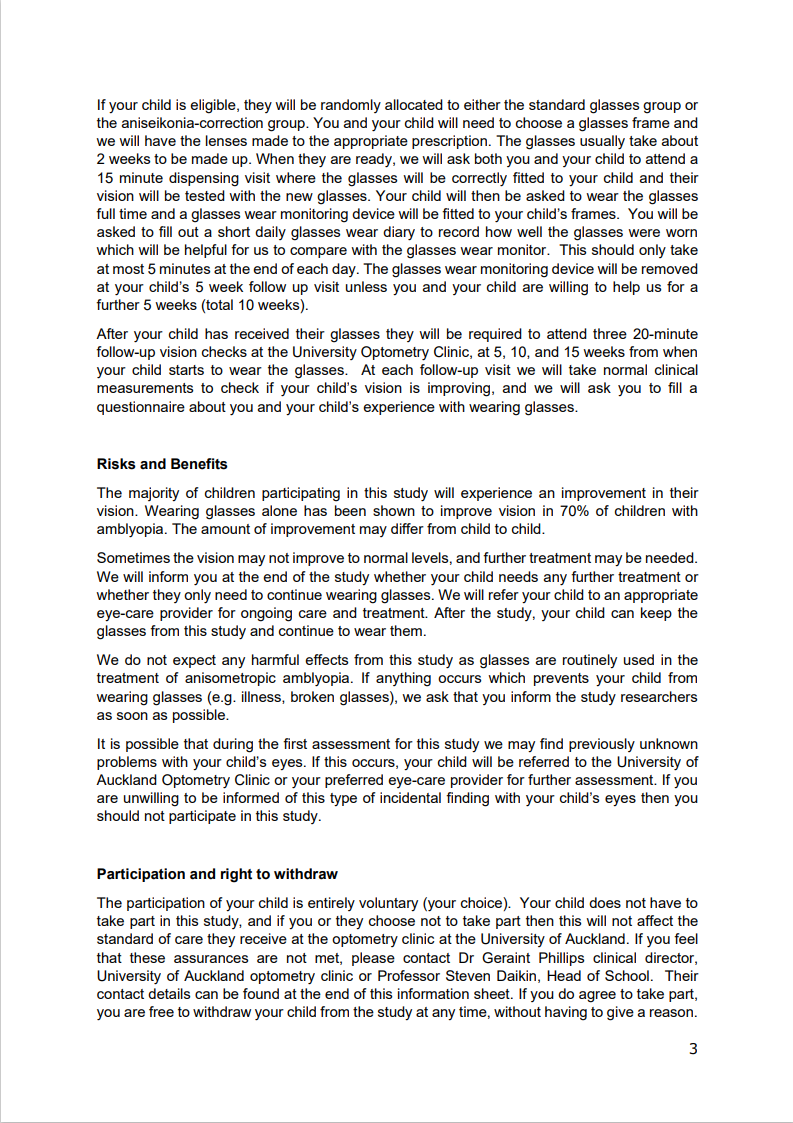


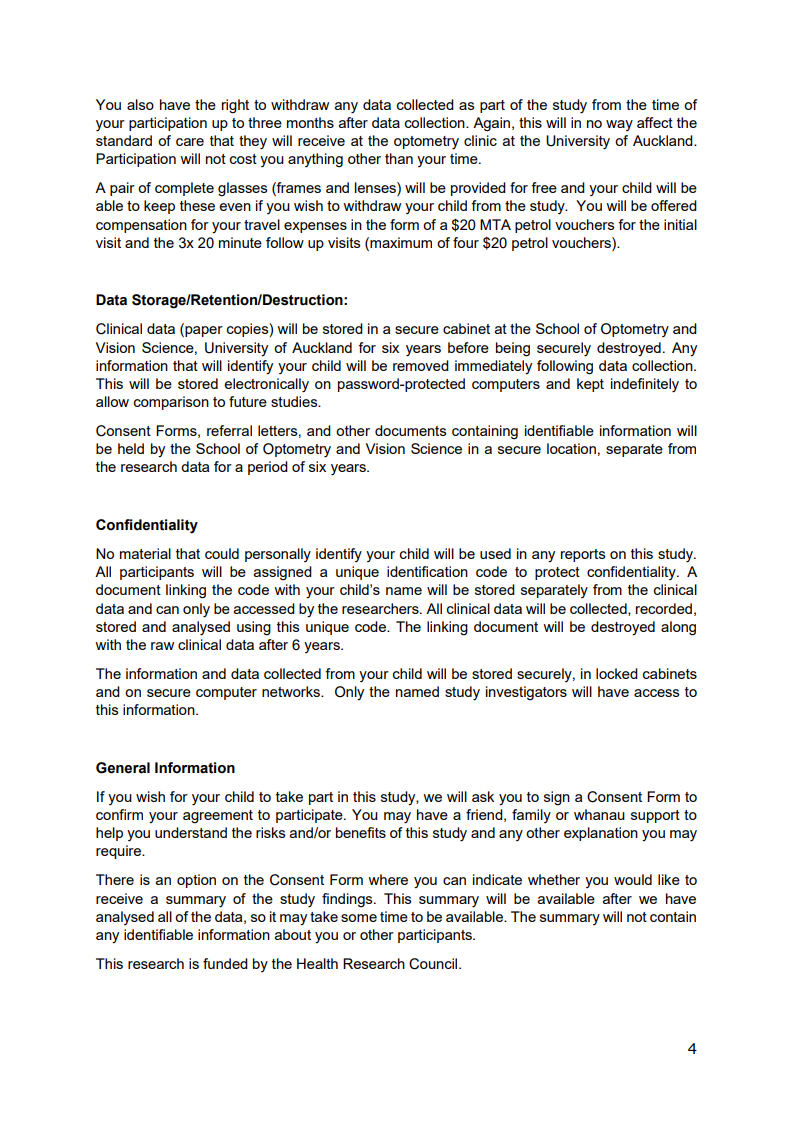


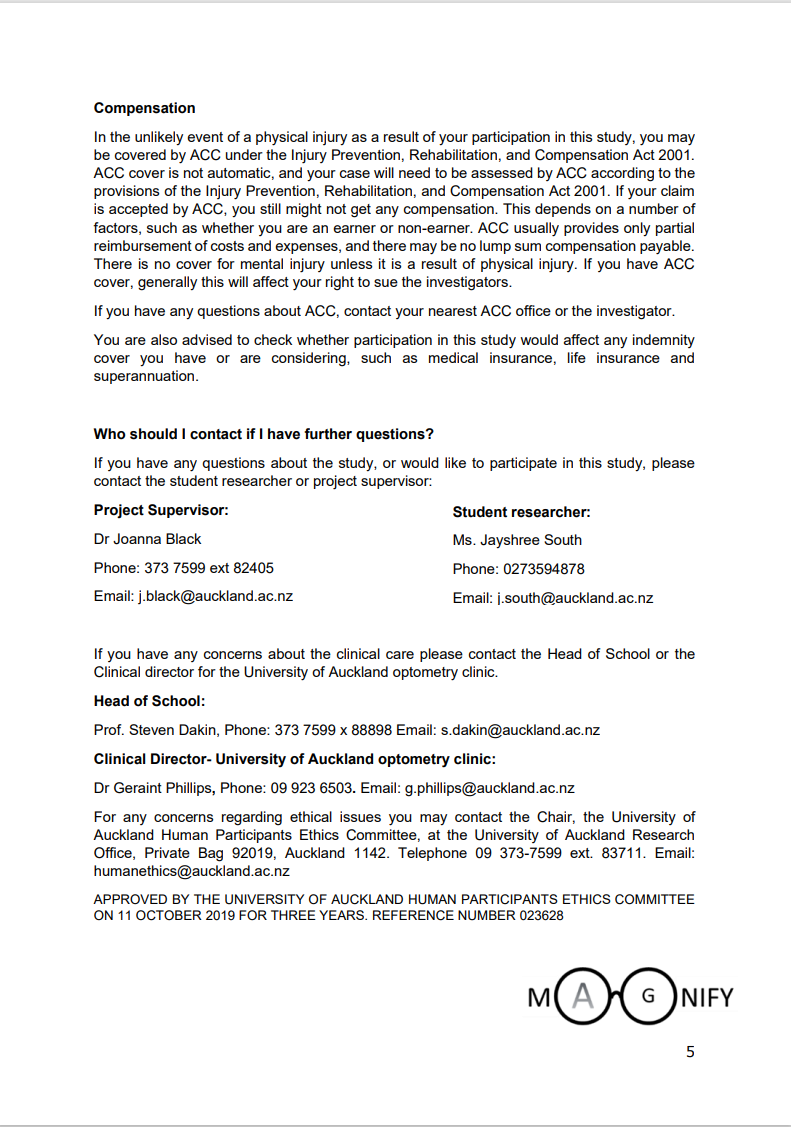


## Child Assent Form


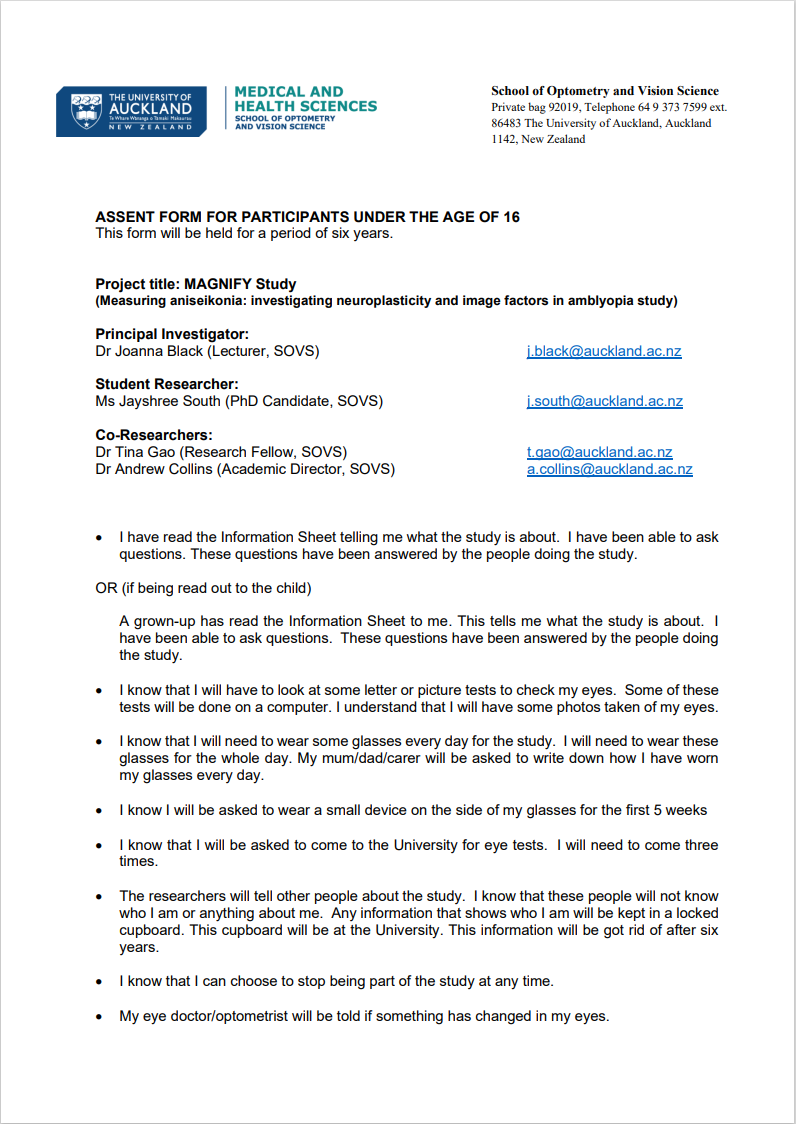


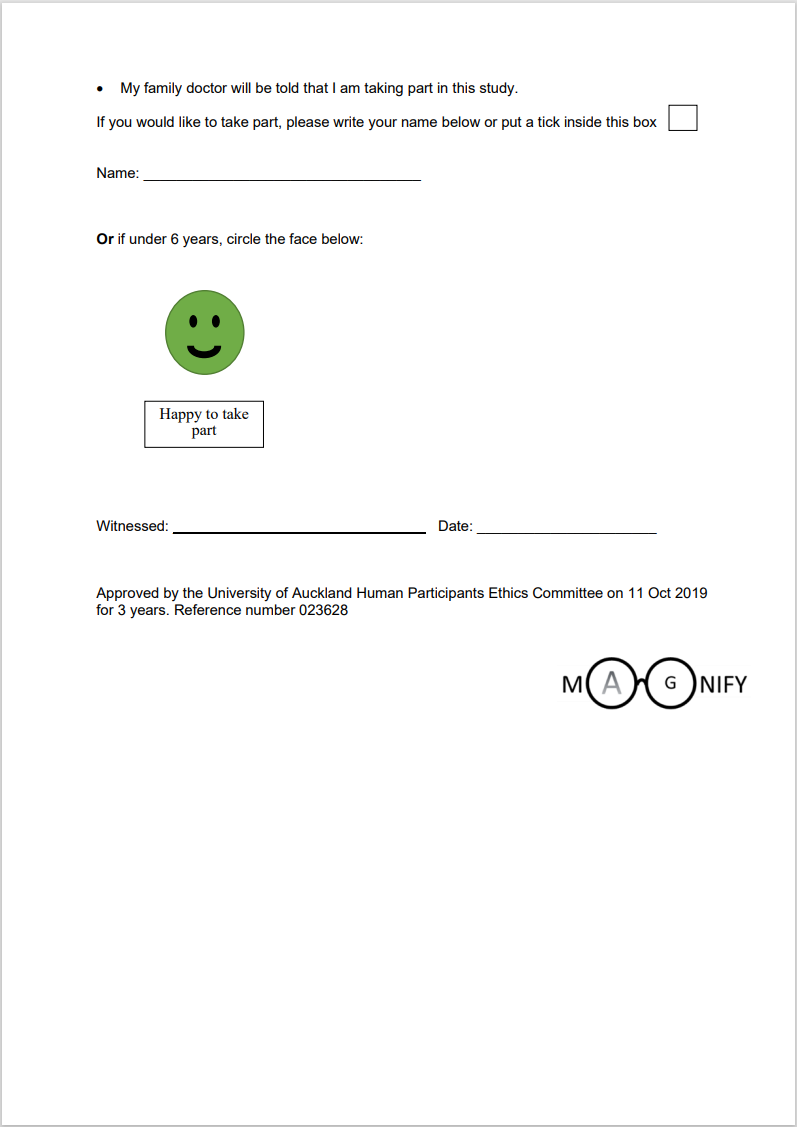


## Child Participant Information Sheet


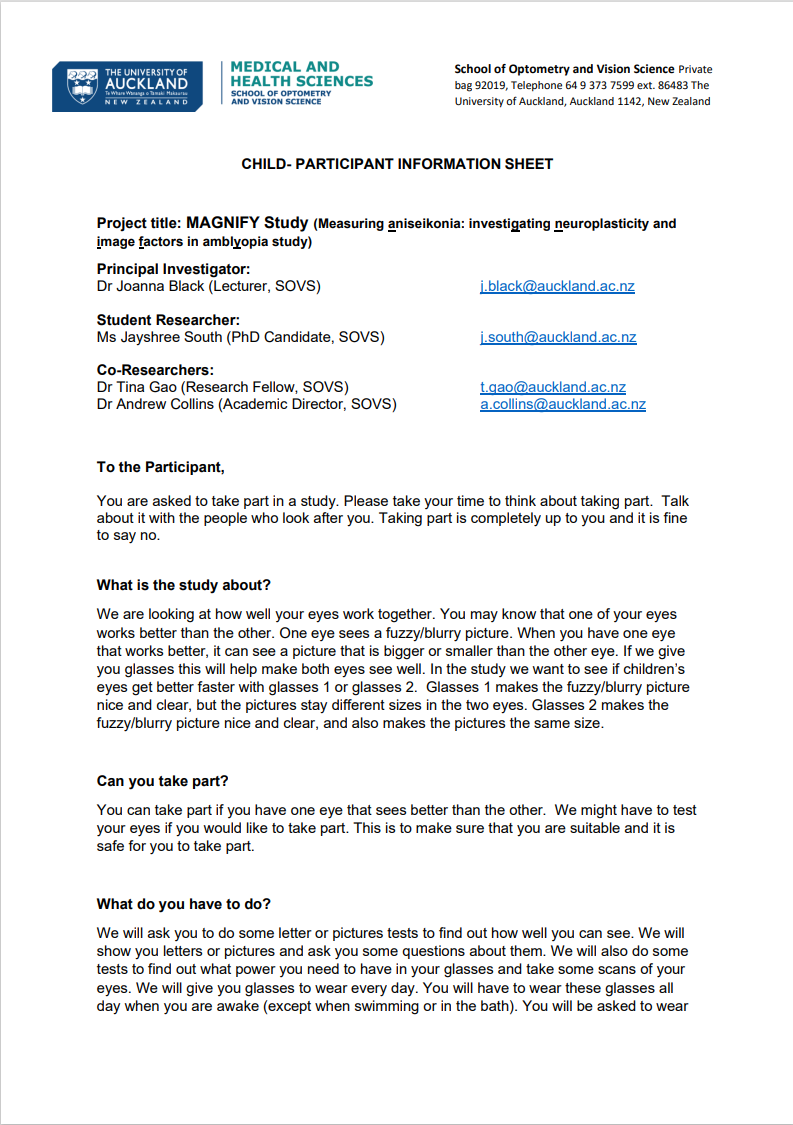


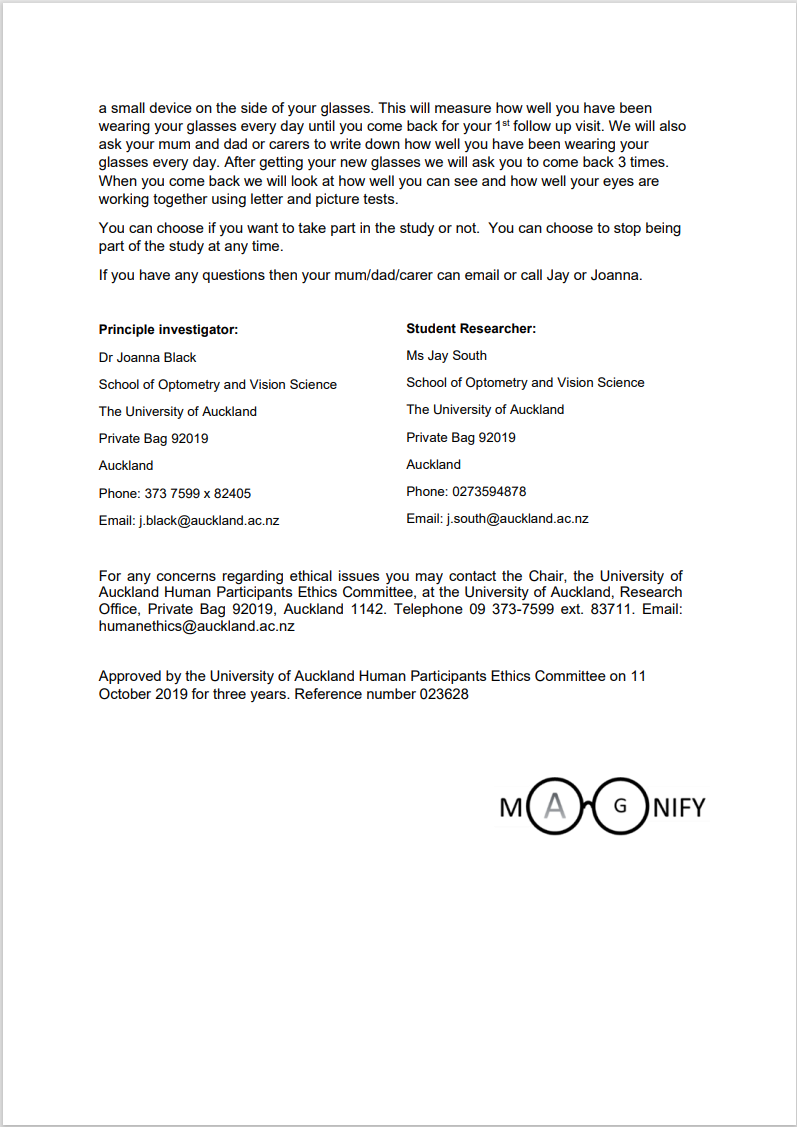


| Trial Registration Data Set | |
| --- | --- |
| Data category | Treatment |
| Primary registry and trial identifying number | Australia New Zealand Clinical Trials Registry. ACTRN12620000061932 |
| Date of registration in primary registry | 24/1/2020 |
| Secondary identifying numbers | None |
| Source(s) of monetary or material support | Health Research Council of New Zealand |
| Primary sponsor | Health Research Council of New Zealand |
| Secondary sponsor(s) | none |
| Contact for public queries | Mrs Jayshree South  School of Optometry and Vision Science The University of Auckland 85 Park Road, Grafton Auckland 1023  New Zealand  **Phone:** +64 273594878  **Email:** J.south@auckland,ac,nz |
| Contact for scientific queries | Dr Joanna Black  School of Optometry and Vision Science The University of Auckland 85 Park Road, Grafton Auckland 1023  **Country**  New Zealand  **Phone:** +64 99232405  **Email:** j.black@auckland.ac.nz |
| Public title | Effect of standard versus customised spectacle treatment for children with asymmetric refractive error |
| Scientific title | Measuring aniseikonia: investigating neuroplasticity and image factors in amblyopic children using aniseikonia-correcting spectacles (MAGNIFY) study |
| Countries of recruitment | New Zealand |
| Health condition(s) or problem(s) studied | Anisometropia, Aniseikonia, Amblyopia |
| Intervention(s) | Active comparator: Aniseikonia correction lenses (spectacle lenses are designed to equalise focus and also reduce aniseikonia (image size differences) between the two eyes |
|  | Placebo comparator: Standard Spectacle lens (spectacle lens which corrects image focus only) |
| Key inclusion and exclusion criteria | Ages eligible for study: 4 to 8 years old Sexes eligible for study: both Accepts healthy volunteers: no |
|  | Inclusion criteria:  • 4-8 Years of age • Anisometropia equal to or greater than 1.50 DS difference in spherical equivalent between eyes • Uncorrected Vision in the worst eye of 6/12 (0.30 logMAR) or worse with an interocular difference of 2 lines or more. Visual acuity will be measured using the ATS-HOTV protocol on the Electronic Visual Acuity tester (EVA). • No manifest strabismus at near or distance on cover test • Refractive corrections meet the following criteria and are based on a cycloplegic refraction that is not more than six months old. The criteria are: 1) Hyperopia: not be under-corrected by more than +1.50D spherical equivalent and the reduction in plus sphere must be identical between the two eyes. 2) Anisometropia: full correction of the anisometropic difference. 3) Astigmatism: full cylindrical power will be prescribed. 4) Cylinder axis in the spectacle lenses in both eyes must be equal to or less than +/-6 degrees of the axis of the cycloplegic refraction when cylinder power is equal to or greater than 1.00 D. • Willing and being able to provide written informed consent for participation in the study |
|  | Exclusion criteria: Presence of a constant or alternating strabismus at any distance, previous amblyopia treatment, Myopia > 6.00D, previous intraocular surgery, any co-existent ocular pathology and any known neurological conditions. Not willing to wear spectacle refractive correction |
| Study type | Interventional |
|  | Allocation: Randomised controlled trial.  Allocation concealment: Participants will be randomized to the standard lenses or aniseikonia-correcting lenses group after they are consented to take part and confirmed to be eligible for the trial. Randomization will be conducted using an envelope or computer-based system. Allocation to each group will be made using a 1:1 ratio |
|  | Primary purpose: Treatment |
|  |  |
| Date of first enrolment | Jan 2020 |
| Target sample size | 50 |
| Recruitment status | Recruiting |
| Primary outcome(s) | Change in best-corrected distance visual acuity in the amblyopic eye from baseline (dispensing visit) to 15 weeks post-randomisation, measured using the highly standardised HOTV protocol EVA testing system (Time frame: From baseline (Dispensing visit) to 15 weeks post-randomisation) |
| Key secondary outcomes | Change from baseline (dispensing visit) in best-corrected distance visual acuity in the amblyopic eye, fellow eye and both eyes at 5, 10 and 15 weeks analysed post-randomisation as a composite score measured using the highly standardised HOTV protocol EVA testing system (time frame: From baseline to 5-, 10- and 15-weeks post-randomisation)  Change from baseline in stereopsis at 5-, 10- and 15-weeks post-randomisation measured using Randot Preschool Stereotest (Baseline measurements from dispensing visit and 5, 10 and 15 weeks after randomization to standard lenses group or aniseikonia correcting lenses group)  Spectacle wear compliance will be based on the total time the participants wore their glasses as recorded in the participants daily wear diary. A participant is considered compliant if they have worn their glasses for equal to or greater than 75% of their awake time (At follow up visits 5, 10 and 15 weeks after randomization)  Change in quality of life using the PedEyeQ Quality of life questionnaire. (Baseline measurement (Dispensing visit) and 15 weeks post randomization to standard lenses or aniseikonia correcting lenses group)  Serious adverse events such as eye strain or headaches, reported by the participants or identified via eye exams during follow-up visits. (5, 10 and 15 weeks after randomization) |
